# Disrupted-in-schizophrenia1 *(DISC1)* L100P mutation alters synaptic transmission and plasticity in the hippocampus and causes recognition memory deficits

**DOI:** 10.1186/s13041-016-0270-y

**Published:** 2016-10-12

**Authors:** Lin Cui, Wei Sun, Ming Yu, Nan Li, Li Guo, Huating Gu, Yu Zhou

**Affiliations:** 1 Department of Physiology, Medical College of Qingdao University, 403 Boya Bldg., 308 Ningxia Rd., Qingdao, Shandong 266071 China; 2 Department of Pathology, Qingdao Municipal Hospital, Affiliated to Medical College of Qingdao University, Qingdao, Shandong 266071 China; 3 Departments of Medicine, Shandong Liming Polytechnic Vocational College, Jinan, Shandong 250116 China

**Keywords:** *DISC1*, L100P mutant, Object recognition, Hippocampus, Synaptic transmission, Synaptic plasticity, Ca^2+^ image

## Abstract

Disrupted-in-schizophrenia 1*(DISC1)* is a promising candidate susceptibility gene for a spectrum of psychiatric illnesses that share cognitive impairments in common, including schizophrenia, bipolar disorder and major depression. Here we report that *DISC1* L100P homozygous mutant shows normal anxiety- and depression-like behavior, but impaired object recognition which is prevented by administration of atypical antipsychotic drug clozapine. Ca^2+^ image analysis reveals suppression of glutamate-evoked elevation of cytoplasmic [Ca^2+^] in L100P hippocampal slices. L100P mutant slices exhibit decreased excitatory synaptic transmission (sEPSCs and mEPSCs) in dentate gyrus (DG) and impaired long-term potentiation in the CA1 region of the hippocampus. L100P mutation does not alter proteins expression of the excitatory synaptic markers, PSD95 and synapsin-1; neither does it changes dendrites morphology of primary cultured hippocampal neurons. Our findings suggest that the existence of abnormal synaptic transmission and plasticity in hippocampal network may disrupt declarative information processing and contribute to recognition deficits in *DISC1* L100P mutant mice.

## Introduction

Major neuropsychiatric illnesses such as schizophrenia, bipolar disorder, major depression and autism spectrum disorder are genetically complex but share not only overlapping symptoms (for example, cognition deficits in learning, memory and attention) and environmental risk factors (for example, influenza, trauma and stress), but also molecular etiology [1]. In particular, disrupted-in-schizophrenia 1 (*DISC1*) gene has been identified as one of the putative susceptibility genes to schizophrenia and other major mental illnesses in multiple pedigrees [2]. *DISC1* was originally discovered in a large Scottish pedigree showing a heavy burden of major psychiatric disorders associated with balanced chromosomal translocation (1:11)(q42.1:q14.3) [3].

There is broad agreement that studying rare, highly penetrant risk mutations, for example *DISC1*, in animal models can not only shed light on the neural integrity of *DISC1* and its relevance to neuropsychiatric disorders, but also help to decipher gene-environment interactions in those illnesses. Quite a few animal models with *DISC1* mutations have reported altered brain morphology, abnormal plasticity, cognitive and affective deficits [4–9]. *N*-ethyl-*N*-nitrosourea (ENU)-induced inheritable missense point mutations in exon 2 of the mouse *DISC1* gene have been of particular interest since Q31L mutant mice showed depression-like behaviors while L100P mutants showed schizophrenia-like phenotypes [4]. *DISC1* L100P and Q31L mutant mice have been considered as potential animal models of psychiatric disorders [10–12]. However, other independent research groups recently reported that no typical schizophrenia-like or depression-like behaviors were observed in *DISC1* L100P or Q31L mutants respectively [13]. This discrepancy may be explained by differences in the genetic background of the mice, the laboratory environment, experimental design and more. A very recent work shows that ENU-generated *DISC1* L100P mice have multiple ENU-induced mutations which sum up to produce the phenotype [14]. Nevertheless, it suggests that further studies are needed to elucidate the cause of behavioral variance associated with *DISC1* L100P or Q31L mutants and to determine whether these mice strains are suitable animal models to study schizophrenia and depression.

In this study, we further analyzed the cognitive and affective behaviors of L100P homozygotes and their WT littermates, and explored the underlying synaptic mechanisms as well. We found that L100P mice show normal affective behaviors but obvious deficits in object recognition which is prevented by clozapine, but not haloperidol. Dentate gyrus (DG) granule cells carrying homozygous mutation of L100P exhibit decreased synaptic excitation and intact synaptic inhibition, meanwhile Schaffer collateral-CA1 synapses of L100P mice display impaired synaptic plasticity. Supportively, both CA1 region and DG of L100P mice hippocampus shows suppression of intracellular [Ca^2+^] elevation after glutamate challenge. However, L100P mutation does not affect expression of the excitatory synaptic markers (PSD-95 and synapsin-1) or dendrites morphology of hippocampal neurons. Our findings thus suggest that the existence of altered synaptic transmission and plasticity may disrupt declarative information processing in the hippocampal network and contribute to recognition deficits observed in *DISC1* L100P mutant mice. Rather normal dendritic structure and excitatory synaptogenesis may correlate to normal affective behaviors observed in L100P mutant mice.

## Methods

### Animals

Male homozygous (L100P/L100P) *DISC1* L100P mutant mice (Disc1 < Rgsc1390>) with C57BL/6 J background were obtained from RIKEN BRC (Tsukuba, Japan, http://www.brc.riken.jp/lab/animal/en/) and then backcrossed to inbred C57BL/6 J female mice from Jackson Laboratory (Stock No 000664, Sacramento, California, USA) for one generation. The resultant heterozygous progeny (L100P/+) were intercrossed to generate L100P/L100P, L100P/+ and +/+ littermates. Mice were group-housed (3–4 mice per cage) in plastic cages after weaning and maintained on a 12 h light/12 h dark cycle with free access to food and water. All animal protocols were approved by the Chancellor’s Animal Research Committee at the University, in accordance with National Institutes of Health guidelines.

### Behavior tests

All behavior analysis was done with 3–6 month-old male L100P homozygotes and their same sex wild-type littermates. All behavioral tests were performed between 9:00 am and 6:00 pm. Animal behaviors were video-tracked and analyzed with Noldus EthoVision XT software.

#### Elevated plus maze (EPM) test

An elevated plus maze test was conducted as described previously [15]. The home-made elevated plus maze consisted of two open arms (29 × 8 cm) and two same-size close arms with 16.5 cm high walls. Four arms were connected by a 5 ×5 cm central square. During test, each mouse was released in the central square of the maze facing one of the close arms and allowed to explore the maze for 5 min. Time spent in the open or close arm; number of arm entries, and total travel distance were calculated.

#### Open field (OF) test

Open field tests were conducted in a square arena (27.3× 27.3× 20.3 cm). Each mouse was released from the center of the arena, total distance traveled, time spend in the center or peripheral area, vertical and stereotyped activity over 10 min were analyzed [15].

#### Novel Object Recognition (NOR) test

Behavior were assessed according to previous publication [9]. Mice were handled 2 min per day for 3 days before training and then were habituated in the empty experimental box (28 × 28 × 25 cm) 10 min per day for another 3 days. During training, mice were placed in the experimental box and exposed to two identical objects for 10 min. One hour later, mice were put back into the same box for the NOR test, during which one object was changed to a new object that the animal have never met. To avoid preference of mice to object A or B, the objects used during training and test were counterbalanced so that half mice in each group were trained with two identical objects A (old object) and the object B was the new object for test, while the other half mice in each group were trained with two objects B (old object) and the object A was the new object for test. The total time mice spent exploring the objects (i.e. sniffing the objects within close proximity), and the percent time mice spent with the old or the novel object was analyzed during a 5- min test.

#### Object-Place Recognition (NPR) test

Mice were handled and habituated in the experimental box (28 × 28 × 25 cm) in the same way as in NOR test. The experimental box for NPR test included a prominent cue on one of the walls [9]. During training, mice were placed in the same box, exposed to two identical objects and were allowed to explore for 10 min. Twenty-four hours after training, mice were placed back into the experimental box with the same two objects: one object stayed in the same location as during training (old location), while the other object was moved to a new location (new location). The new location of the object was counterbalanced so that half of the mice in each group saw the object in the new location on the left side, and the other half saw it on the right side. The total time mice spent exploring the objects (i.e. sniffing the objects within close proximity), and the percent time mice spent with the object in old location or the object in new location was analyzed during a 5-min test.

#### Social interaction (SI) test

Social behaviors were assessed according to previously described methods with minor modifications [14]. The behavioral test consisted of two sessions: habituation and sociability. In habituation, the testing mouse was placed in the experimental box (30 × 60 cm ×25 cm) and allowed to freely explore for 5 min. In social interaction session following habituation, testing mouse was put back into the same experimental box and introduced to an unfamiliar, ovariectomized, 3-6month-old C57BL/6 J female mouse enclosed in the wire-bar cup in the center of the experimental box. The testing mouse was allowed to freely explore the box for 10 min. The social interaction (i.e. sniffing the female mouse within close proximity) time and frequency is recorded for 10 min.

#### Water maze training and spatial memory test

Water maze was conducted as previously described [16]. A10-cm-in-diameter escape platform located in one of the 4 quadrant was fixed 0.5 cm underneath the water surface. The pool was surrounded by curtains with distinct cues hung on them. Mice were handled for 7 days before training and trained with six trials presented in two blocks (inter-block interval 2 h, inter-trial interval 30 s) per day for 6 days. During each trial mice were released into the pool, facing the wall, from one of 6 pseudo-random start locations and given 60 s to find the hidden platform. Spatial memory was assessed immediately after completion of the 3^rd^ day, 5^th^ day’s training and 24 h after the 6^th^ day’s training. During probe test, the platform was removed and the mouse was allowed to search for 60 s in the pool. The percentage of time mice spent in each quadrant during the probe test was analyzed.

#### Forced swimming (FST) test

Mice were gently released into a transparent plastic cylinder (25 cm height ×10 cm diameter) filled with water (24.5 ± 0.5 °C) up to a depth of 15 cm for 5 min. The water surface was 10 cm below the top of cylinder. The total immobility time and the latency to first immobility were analyzed [9].

### Primary hippocampal neuron culture

Trypsin-dissociated hippocampal neurons derived from 18-day embryo (E18) were cultured in Neurobasal medium with B27 supplement according to previous report [17]. Briefly, E18 embryos were removed from maternal mice anesthetized with diethyl ether. Hippocampus from individual embryo was quickly dissected and submerged into a 12-well plate filled with Ca^2+^- and Mg^2+^-free HEPES-buffered Hank’s balanced salt solution (pH 7.45), followed by a digestion with 0.25 % w/v trypsin; Meanwhile some tissue from the same embryo was frozen for later genotyping analysis. After trituration through a Pasteur pipette, neurons were centrifuged (1000 g for 5 min) and re-suspended in Neurobasal medium containing 2 % B27 serum-free supplement, 1 % v/v penicillin/streptomycin, 0.5 mM glutamine, and 10 mM glutamate (Sigma). Dissociated cells were then plated at a density of 0.03 × 10^6^ cells/cm^2^ onto either Fisher Brand round 12-mm-diameter coverslips (for patch-clamp recording and immunostaining) or confocal microscopy dishes (for calcium image), and at a density of 0.05 × 10^6^ cells/cm^2^ to 6-well plates (for Western blot). Dishes, coverslips and plates were pre-coated with poly-D-lysine (50 μg/ml, Sigma). Cultures were kept at 37 °C in a 5 % CO2 humidified incubator. One third to half of the medium was replaced twice a week with Neurobasal culture medium containing 2 % B27 supplement and 0.5 mM glutamine. All culture reagents were ordered from Invitrogen unless specified otherwise.

### Immunofluorescence staining

Immunofluorescence staining was carried out as described previously [17]. Briefly, cultured neurons (DIV15) on coverslips were fixed by 4 % paraformaldehyde (PFA) plus 4 % sucrose in phosphate-buffered saline (PBS, PH 7.4) for 20 min and permeabilized by 0.2 % Triton X-100 for 10 min. After blocked with 10 % normal donkey serum (Jackson Immuno Research) for at least 2 h, neurons were incubated with primary antibodies, including mouse anti-PSD95 (1:500, Millipore), goat anti-synapsin-1 (1:500, Abcam) and goat anti-MAP-2 (1:500, Millipore) in 3 % NDS for 1 h. After washing with PBS for three times, neurons were incubated in 3 % NDS containing Alexa Fluor 568 goat anti-mouse IgG (1:1000, Invitrogen) or Alexa Fluor 405 goat anti-rabbit IgG (1:1000, Invitrogen) for another 1 h. After washing with PBS for three times, the coverslips were incubated with 4′, 6-diaminodino-2-phenylindole (DAPI, Invitrogen, 1:2,000) for 15 min before mounted with ProLong® Gold Antifade Reagent (Invitrogen). Fluorescent images were acquired with a laser-scanning confocal microscope (LSCM510 Meta, Zeiss) and a 63 ×oil-immersion objective lense. Gain, threshold, and black levels were not changed during the individual experiment. Neuronal images were analyzed using MetaMorph and customized filter sets. All image analysis was done blind to the experimental conditions.

Spines were defined as dendritic protrusions of 0.5–3 μm length, with or without a head. Excitatory synapse density was measured by counting the number of PSD95-positive spines (green) co-localized with synapsin-1-postive boutons (red) per 100 μm dendritic length (including secondary and tertiary dendrites) per neuron [18]. Co-localization of two fluorescent signals was determined using “colocalization” module in MetaMorph as described [19].

### Protein isolation and western blotting

Synaptosome (SS)- and PSD-enriched fractions were prepared from adult mice (3 to 4 months old) with a protocol described in our previous publication [16]. In brief, the forebrains were rapidly dissected and homogenized in ice-cold homogenization buffer containing 4 mM HEPES-NaOH (pH 7.4), 0.32 M sucrose and fresh protease inhibitor mixture (Roche). After centrifugation, 2 ml supernatant (S1) was diluted with the same volume of 10 % Percoll (GE Healthcare Bio-Sciences) and laid on top of a 10 and 20 % discontinuous Percoll gradient. The interface between 10 and 20 % Percoll was collected after centrifugation (33,000 × g for 5 min at 4 °C) and diluted with PSD buffer (PSDB) containing 40 mM HEPES-NaOH (pH 8.1) and fresh protease inhibitor mixture. After centrifugation at 4 °C for 20 min (20,000 × g), the SS pellet was re-suspended thoroughly in PSDB. Then, 0.5 % Triton X-100 was added to SS lysates to enhance solubilization. After stirring for 15 min at 4 °C and centrifugation for 40 min (40,000 × g) at 4 °C, the final pellet (PSD) was re-suspended in PSDB and stored at −80 °C for use. Protein concentrations were determined with a BCA assay kit (Thermo).

Equal amounts of SS and PSD protein were separated by electrophoresis on a 4–12 % SDS-PAGE gel (Invitrogen) and then transferred to nitrocellulose membranes. After blocking with 5 % (w/v) nonfat dry milk in TBS-T (Tris-buffer saline containing 0.1 % Tween-20) for 1 h at room temperature, membranes were hybridized with a primary antibody of interest overnight at 4 °C. After washing with TBS-T, membranes were incubated with a secondary antibody in TBS-T containing 5 % nonfat milk for 1 h at room temperature. Signals were visualized by ECL (Thermo). Exposure time was adjusted so that the signals measured were in a linear range. The following primary antibodies were used: PSD-95 (1:2000, Millipore), synapsin-1 (1:1000, Abcam), synaptophysin (SynPhy, 1:2000, Millipore), αCaMKII (1:2000; Millipore), β-actin (1:1000; Sigma), GluA1 (1:1000, Abcam), GluN2B (1:1000, Abcam), β-tubulin (1:1000, Abcam) respectively. β-tubulin and β-actin were used as controls for protein loading. The protein/β-tubulin (or β-actin) ratio of the wild-type group was used as a standard and L100P group was normalized to it before statistical analysis.

### Hippocampus slices preparation and electrophysiological recordings

Hippocampal slice was prepared according to previous description [16]. Briefly, slice was sectioned in oxygenated ice-cold cutting solution (pH 7.40) containing (in mM) 2.5 KCl, 26 NaHCO_3_, 1 NaH_2_PO4, 7 MgSO_4_, 1 CaCl_2_, 30 Glucose, 119 choline chloride, 3 sodium pyruvate, 1 kynurenic acid and 1.3 sodium L-ascorbate. Coronal slices (400 μm thick) containing hippocampus were cut on a vibratome (VT-1000, Leica, Germany) and transferred to recovery solution containing (in mM) 85 NaCl, 2.5 KCl, 1.25 NaH_2_PO4, 0.5 CaCl_2_, 4 MgCl_2_, 24 NaHCO_3_, 25 glucose and 50 sucrose to recover for 30 min at 30 °C and then at least 1 h at room temperature prior to recording.

Whole cell patch-clamp recordings in voltage-clamp mode were obtained from pyramidal neurons in hippocampal CA1 and granule cells in DG. The glass micropipettes (4–6 MΩ) was filled with internal solution (pH 7.30) containing (in mM) 130 CsMeSO_4_, 10 CsCl, 4 NaCl, 1 MgCl_2_, 5 MgATP, 5 EGTA, 10 HEPES, 0.5 Na_3_GTP, 10 phosphocreatine and 4 QX-314. During recordings, slices were continuously perfused with artificial cerebral spinal fluid (ACSF) containing (in mM) 120 NaCl, 3.5 KCl, 2.5 CaCl_2_, 1.3 MgSO_4_, 1.25 NaH_2_PO_4_, 26 NaHCO_3_ and 10 glucose, at a flow rate of ~2 ml/min at 31–33 °C. Spontaneous inhibitory postsynaptic currents (sIPSCs) were recorded at a holding potential of +20 mV with 3 mM kynuric acid in perfused ASCF; while spontaneous excitatory postsynaptic currents (sEPSCs) were recorded at a holding potential of −70 mV in ASCF with 50 μM AP-5 and picrotoxin in ACSF. Miniature inhibitory and excitatory postsynaptic currents (mIPSCs and mEPSCs) were recorded with the application of 1 μM TTX in external solutions. sEPSCs, mEPSCs, sIPSCs and mIPSCs were analyzed using Mini Analysis Program. Event counts were carried out by an experimenter blind to genotype.

Field excitatory postsynaptic potentials (fEPSPs) were evoked in acute hippocampal slices every 30 s with FHC bipolar platinum electrodes placed in the medial perforant path (MPP) for DG or in Schaffer collateral path (SC) for CA1. For input-output (I/O) experiments measuring evoked basal transmission, synaptic input was the peak amplitude of the fiber volley, and the output was the initial slope of the fEPSP from average of three individual traces. Paired-pulse ratio (PPR) were determined by evoking two fEPSPs (average of three individual traces) that are 10–400 ms apart and dividing the initial slope of the second fEPSP by that of the first (fEPSP2/fEPSP1). The paired pulses were delivered every 20 s. For synaptic plasticity experiments, LTP at SC-CA1 synapses was induced by a single tetanus of 100 pulses at 100 Hz. LTP at MPP-DG synapses was induced by four trains of 100 Hz tetanus with 20 s intervals. All test stimuli and tetanus pulses were 100 μs in duration and 1/2–2/3 of maximal stimulation strength (100 μA). All the recording data were filtered at 1 or 2 kHz and digitized at 10 kHz. Analog to digital conversion was performed using Digidata 1440A (Molecular Devices). Data were acquired using Clampex 10 (Molecular Devices), and analyzed using Clampfit 10 (Axon Instruments). The experimenters were blind to the genotypes of the mice. All the chemicals used in electrophysiological recordings were purchased from Sigma.

### Calcium image

The juvenile mice (P8 ~ P15) were decapitated and brains were rapidly removed and placed in ice cold ACSF composed of (in mM): 124 NaCl, 26 NaHCO_3_, 2 KCl, 1.25 NaH_2_PO4, 2 CaCl_2_, 2 MgSO_4_, and 10 D-glucose. Coronal slices (300 μm thick) containing the hippocampus were prepared and allowed to recover in oxygenated ACSF at room temperature for at least 1 h before loaded with 10 μM Ca^2+^ indicator fluo-4 AM (acetoxymethyl ester of fluo-4, Thermo Fisher Scientific) for 40 min at room temperature. Slices were then transferred to a perfusion chamber and were continuously perfused with ACSF at a rate of ~2.5 ml/min. All solutions were continuously bubbled with 95 % O_2_/5 % CO_2_. The fluorescence imaging was performed with Olympus BX61W1 confocal microscope (Olympus). Fluorescence excited at 488 nm was imaged through a 40× objective lens. For quantitative analysis, multiple cells in the whole field of view were marked as the regions of interest (ROIs). The fluorescence images of same ROIs were collected every second for 400 s and analyzed using Fluoview1000 software (version 3.1a, Olympus). After detection of baseline fluorescence intensity (F0) for 100 s, slices were exposed to 50 μM glutamate to induce a rapid increase in cytoplasmic [Ca^2+^]. Fluorescence intensity after glutamate application (F1) was monitored continuously for 300 s. Glutamate-evoked elevations of intracellular [Ca^2+^] were estimated as ΔF/F0 after background subtraction [20]. Experiments were conducted on 2–4 slices from each hippocampus and 3–5 mice with same genotype.

### Data analysis

Results were expressed as mean ± SEM. Data were analyzed using one-sample *t-*test, unpaired *t*-test, one-way ANOVA, two-way ANOVA or two-way repeated measures as appropriate. *P* < 0.05 indicates significant difference between groups. Statistical analysis was performed with GraphPad Prism 5.0 (GraphPad Software).

## Results

### L100P mutant mice showed normal anxiety- and depression-like behaviors

**Fig. 1:**
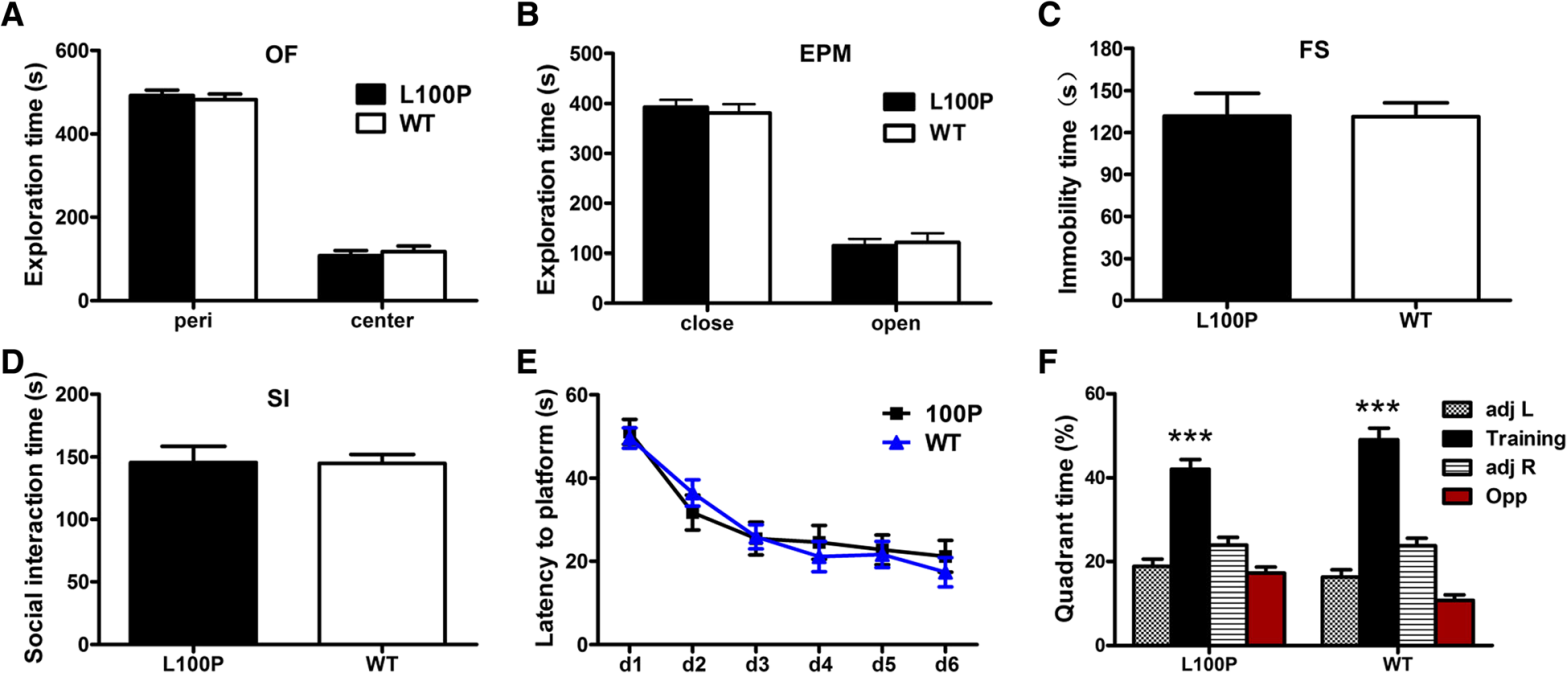
*DISC1* L100P homozygous mutant shows normal behaviors in locomotor activity, anxiety and depression, spatial learning and memory. **a** In the open field (OF) test, L100P mice (*n* = 10) and WT mice (*n* = 11) spent similar time exploring center or peripheral area. **b** In the elevated plus maze (EPM) test, L100P mice (*n* = 10) and WT mice (*n* = 11) spent similar time exploring the open or close arms. **c** In the forced swim (FS) test, the immobility time of L100P mice (*n* = 10) and WT mice (*n* = 11) were similar. **d** In social interaction (SI) test, L100P mice (*n* = 10) and WT mice (*n* = 10) spent similar time interacting with an unfamiliar, ovariectomized C57BL/6 J female mouse. **e** Time to locate the hidden platform as a function of training days in Morris water maze. **f** Water maze probe test at day 5 showing that both L100P mice (*n* = 19) and WT littermates (*n* = 20) spent significant more time searching in the training quadrant where the platform was located during training than in the other three quadrants. Two-way ANOVA and Bonferroni post-tests, ***P < 0.001. All data are shown as means ± SEM.

First, we measured locomotor activity and exploratory behavior of L100P homozygous mutants (L100P) and their littermate controls (WT) in an open field (OF). We found no effect of genotype on total travel distance, vertical activity, and stereotypic counts (data not shown). L100P mice showed identical center or peripheral exploration time compared to WT controls, which indicates normal anxiety (Fig. 1a; two-way ANOVA, genotype *F*_*(1, 38)*_ = 10^−12^, *P* > 0.05). Consistently, in the elevated plus maze (EPM) test, a well-established test to measure anxiety, we did not found significant effect of genotype on the time spent in open or close arms either (Fig. 1b; two-way ANOVA *F*_*(1, 38)*_ = 0.026, *P* > 0.05). To assess depression-like behavior, we measured the immobility during the force swimming (FS) test and found that the total immobility time of L100P mice was comparable to that of WT controls (Fig. 1c; unpaired *t-*test, *P* > 0.05). Since social dysfunction is a significant problem in schizophrenia [21], we also conducted the social interaction (SI) test and found no significant difference between the L100P mutants and WT controls in their social interaction time (Fig. 1d; unpaired *t*-test, *P* > 0.05). Therefore, in our study, we did not found increased anxiety- or depression-like behaviors in L100P homozygous mutants, a finding that is consistent with recent report [13].

### L100P mutant mice showed object recognition memory deficits

**Fig. 2:**
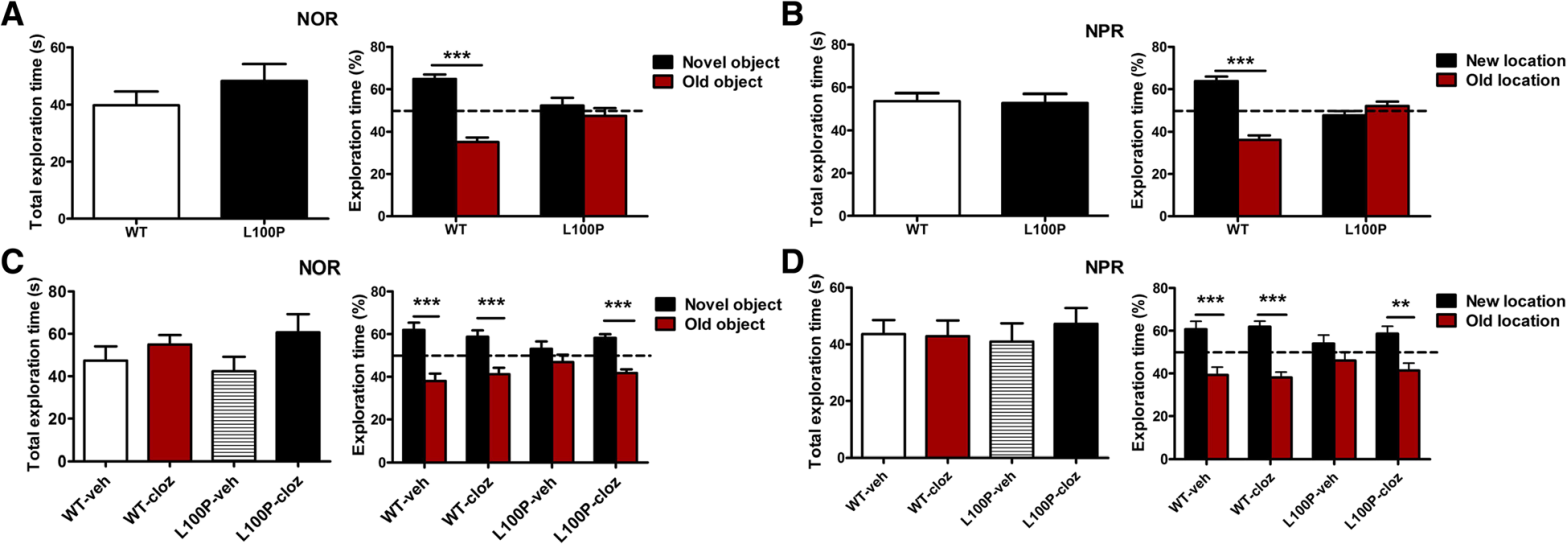
*DISC1* L100P homozygous mutant shows impaired object recognition memory which is reversed by Clozapine. **a** Novel objects recognition (NOR) test. *Left*, L100P mutants and WT mice spent similar time exploring the two objects during test. *Right*, the percentage of exploration time during NOR test at 1 h after training. WT mice (*n* = 20) spent significantly more time exploring the novel object versus the old object; while L100P mice (*n* = 19) did not. Two-way ANOVA and Bonferroni post-tests, ***P < 0.001. **b** Object-place recognition (NPR) test. *Left,* L100P mutants and WT mice spent similar time exploring the two objects during test. *Right*, the percentage of exploration time during NPR test at 24 h after training. WT mice (*n* = 21) spent significantly more time exploring the object at the new location versus the object at the old location; while L100P mice (*n* = 19) did not. Two-way ANOVA and Bonferroni post-tests, ***P < 0.001. **c** Clozapine treatment rescued new object recognition deficits in L100P mice. *Left*, total objects exploration time in NOR test. *Right*, the percentage of exploration time during NOR test at 1 h after training. L100P mice treated with clozapine (L100P-cloz, *n* = 10) spent significantly more time exploring the novel object versus the old object, while L100P mice received vehicle administration (L100P-veh, *n* = 9) did not. Two-way ANOVA and Bonferroni post-tests, ***P < 0.001. WT-veh *n* = 10, WT-cloz *n* = 10. **d** Clozapine treatment rescued object-place recognition deficits in L100P mice. *Left*, total objects exploration time in NPR test. *Right*, the percentage of exploration time during NPR test at 24 h after training. L100P mice treated with clozapine (L100P-cloz, *n* = 10) spent significantly more time exploring the object at the new location versus the object at the old location, while L100P mice received vehicle administration (L100P-veh, *n* = 7) did not. Two-way ANOVA and Bonferroni post-tests, **P < 0.01 and ***P < 0.001. WT-veh *n* = 8, WT-cloz *n* = 10. All data are shown as means ± SEM.

Cognitive deficits are commonly associated with major neuropsychiatric illnesses and have been reported in *DISC1* mutations [4–7, 9, 22]. To evaluate whether our L100P mutant still shows abnormality in learning and memory without anxiety or depression phenotypes, L100P homozygotes and WT controls were tested in novel object recognition (NOR) and object-place recognition task (NPR), two tasks sharing many of the same motivational and visual-perceptual demands excepting that the latter is considered to be heavily dependent on hippocampus [23]. We found that L100P mice showed profound deficits in both NOR and NPR tests: while WT mice spent significantly more percentage of time exploring the novel object in NOR test (Fig. 2a; two-way ANOVA, *F*_*(1 ,74)*_ = 33.67; Bonferroni post-tests, novel object versus old object, *t* = 7.15, *P* < 0.001) or the object at the new location in NPR test (Fig. 2b; two-way ANOVA, *F*_*(1,74)*_ = 30.97; Bonferroni post-tests, new location versus old location, *t* = 9.50, *P* < 0.001), the L100P mutants spent similar time exploring two objects in both NOR test (Fig. 2a; two-way ANOVA, *F*_*(1, 74)*_ = 33.67; Bonferroni post-tests, novel object versus old object, *t* = 1.13, *P* > 0.05) and NPR test (Fig. 2b; two-way ANOVA, *F*_*(1, 74)*_ = 30.97; Bonferroni post-tests, new location versus old location, *t* = 1.49, *P* > 0.05). Importantly, L100P mutants and WT mice spent similar time to explore two objects in NOR (Fig. 2a) and NPR tests (Fig. 2b), indicating that the recognition memory deficits observed in L100P mutants were not caused by lack of exploration activity or anxiety.

To assess whether L100P mutants had spatial memory deficits, mice were trained and tested in Morris water maze. Probe test showed that L100P mutants acquired stable spatial memory same as the WT mice: There was no effect of genotype on the percentage of quadrant searching time (two-way ANOVA, *F*_*(1, 144)*_ = 0.135, *P* > 0.05); both groups of mice showed strong spatial bias for the quadrant where the platform was located during training (Fig. 1f; two-way ANOVA, *F*_*(3, 144)*_ = 108.3, *P* < 0.001; Bonferroni post-tests, target quadrant versus others, *P* < 0.001). In addition, L100P and WT mice required similar time to locate the hidden platform during training (Fig. 1e; two-way ANOVA, *F*_*(1, 222)*_ = 0.15, *P* > 0.05), indicating that spatial learning of L100P mice were normal. Therefore, we reported here that L100P homozygous mutant shows impaired object recognition memory while relatively normal spatial memory.

### Clozapine treatment reversed the recognition deficits in L100P mutant mice

To test whether antipsychotics treatment could reverse the recognition deficits observed in L100P mutants, antipsychotics were administered by i.p. injection 40 min before both training and test. Our analysis showed that the atypical antipsychotic clozapine (0.6 mg/kg dissolved in 10 % DMSO, Tocris), an antagonist of both dopamine and serotonin receptors, prevented the memory deficits of L100P mutants in novel object recognition (NOR, Fig. 2c) and object-place recognition (NPR, Fig. 2d). In comparison to the mutant group treated with vehicle (L100P-veh) that spent the same percentage of time exploring the new object (or the object at the new location) compared to the old object (or the object at old location), the L100P group treated with clozapine (L100P-cloz), the WT vehicle groups (WT-veh) and the WT clozapine groups (WT-cloz) all spent significantly more time exploring the novel object (Fig. 2c; two-way ANOVA, *F*_*(1, 70)*_ = 58.18, *P* < 0.001; Bonferroni post-tests, *P* < 0.001, novel object versus old object) during NOR test or the object at the new location during NPR test (Fig. 2d; two-way ANOVA, *F*_*(1, 62)*_ = 52.86, *P* < 0.001; Bonferroni post-tests, *P* < 0.01 or *P* < 0.001, object at the new location versus object at the old location). Remarkably, during training, all four groups of mice spent comparable time exploring the two objects, indicating that clozapine treatment at the relative low dose of 0.6 mg/kg did not affect exploratory behavior (Fig. 2c and d; one-way ANOVA, *F*_*(3,35)*_ = 1.39 for NOR and *F*_*(3,31)*_ = 0.21 for NPR, *P* > 0.05). Interestingly, similar treatment with the typical antipsychotic haloperidol, a dopamine D2 receptor antagonist, could not reverse the recognition deficits observed in L100P mutants (data not shown).

### L100P mutant mice showed abnormal synaptic transmission and plasticity in hippocampal network

**Fig. 3:**
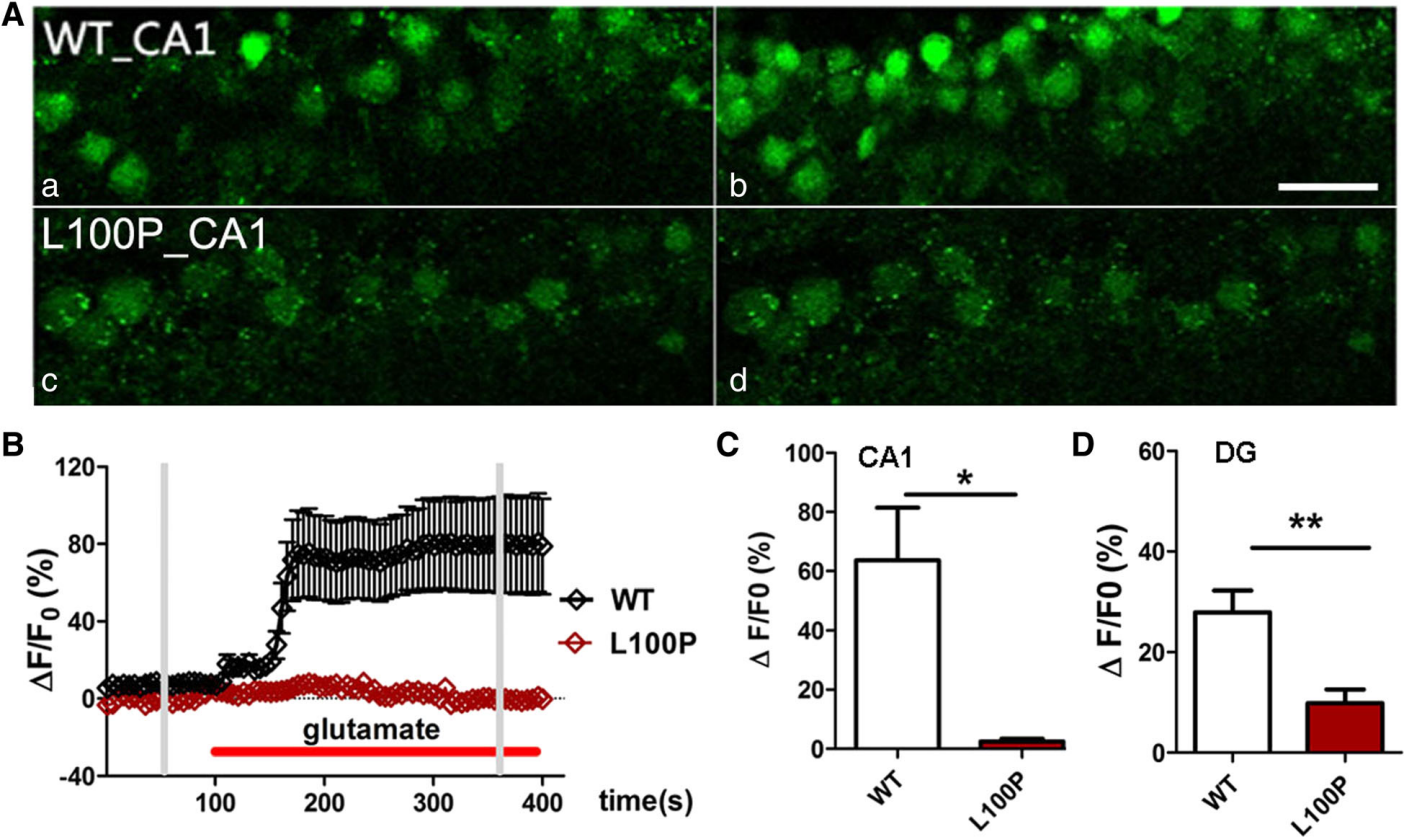
Ca^2+^-image study reveals suppression of glutamate-evoked elevation of cytoplasmic [Ca^2+^] in L100P mutant hippocampus. **a** Representative confocal images of CA1 pyramidal cells loaded with Fluo-4 AM (*green*) before (a, c) and during (b, d) perfusion with 50 μM glutamate. Bar 50 μm. **b** Time course of glutamate-induced intracellular [Ca^2+^] increase in CA1 pyramidal neurons. Fluorescence intensity changes ΔF was background corrected and normalized to the resting fluorescence (F0). The addition of glutamate was indicated by the horizontal bar (*red*). The grey vertical bars indicate the selective time points that images shown in A were taken. Traces represent averaged responses of selected cells in the same field of view. **c** Summary of glutamate-induced [Ca^2+^] elevations in CA1 pyramidal neurons of juvenile mice (P8 ~ P15). WT, *n* = 31 cells from 4 mice and L100P, *n* = 25 cells from 3 mice, * *P* < 0.05, unpaired *t*-test. **d** Summary of glutamate-induced [Ca^2+^] elevations in DG granule cells of juvenile mice (P8 ~ P15). WT, *n* = 22 cells from 4 mice and L100P, *n* = 15 cells from 3 mice, **P < 0.01, unpaired *t*-test. Data were averaged during 200–300 s after the onset of perfusion with glutamate, and normalized by the mean fluorescence intensity obtained during the baseline period (0–100 s) before glutamate perfusion for each cell. All data are shown as means ± SEM.

**Fig. 4:**
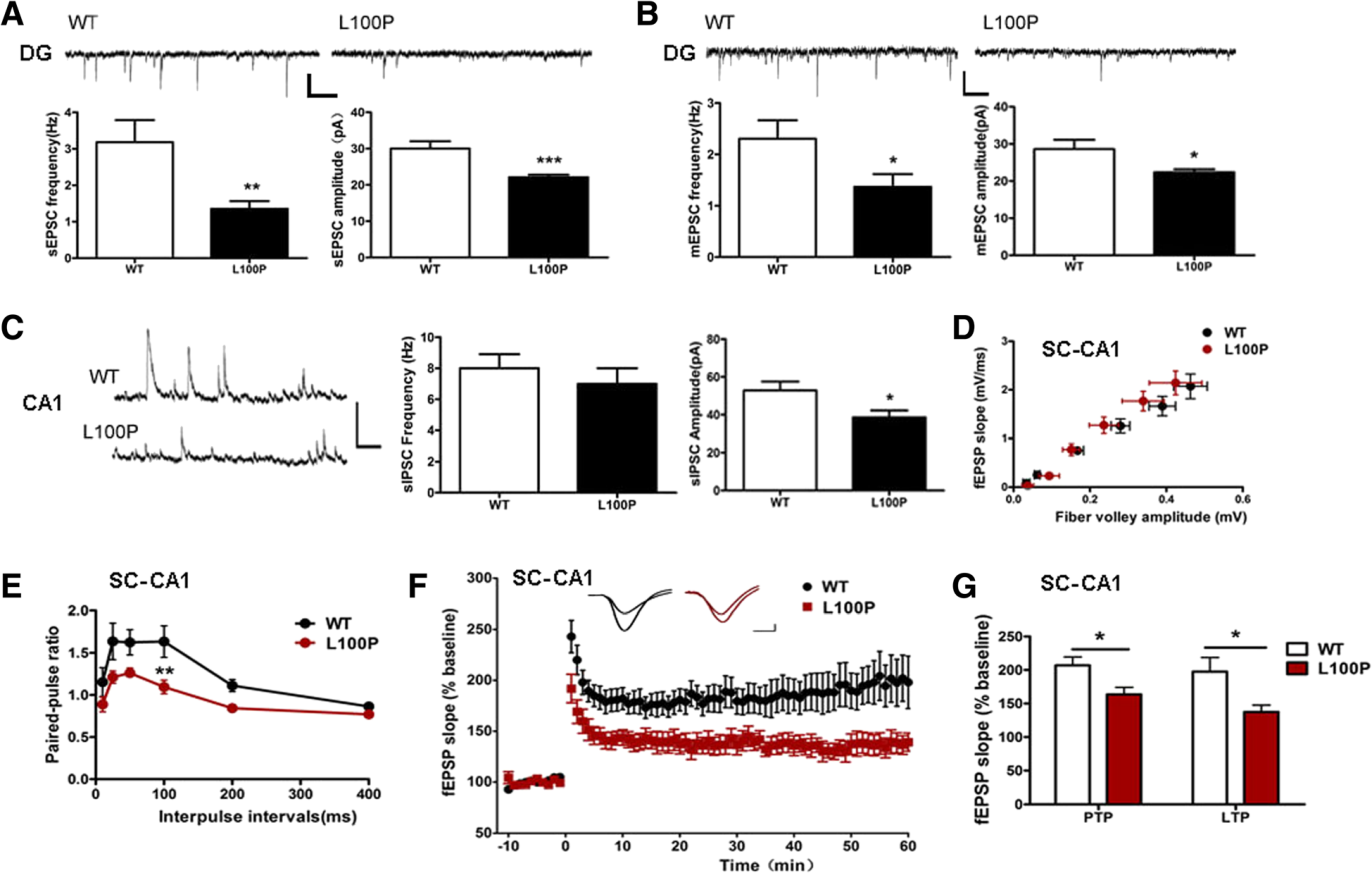
Synaptic transmission and plasticity are impaired in L100P mutant hippocampus. **a** sEPSCs in dentate granule cells. *Top*, sample sEPSCs traces recorded in L100P (right) and WT (left) granule cells. *Bottom*, summary of sEPSCs frequency (*left*) and amplitude (*right*) showing reduced sEPSCs frequency and amplitude in L100P granule cells compared to the WT controls. **P < 0.01 or *** *P* < 0.001, unpaired *t*-test, *n* = 9 (5 mice) for WT group and *n* = 11 (5 mice) for L100P group. Scale bars, 20 pA and 200 ms. **b** mEPSCs recorded in dentate granule cells. *Top*, sample mEPSCs traces recorded in L100P (*right*) and WT (*left*) granule cells. *Bottom*, summary of mEPSCs frequency (*left*) and amplitude (*right*) showing reduced mEPSCs frequency and amplitude in L100P granule cells compared to the WT controls. *P < 0.05, unpaired *t*-test, *n* = 10 (5 mice) for WT group and n = 11 (5 mice) for L100P group. Scale bars, 20 pA and 200 ms. **c** sIPSCs in CA1 pyramidal cells. *Left*, sample sIPSCs traces recorded in L100P (*bottom*) and WT (*top*) pyramidal cells. Summary of sIPSCs frequency (*middle*) and amplitude (*right*) showing reduced sIPSCs amplitude but normal frequency in L100P cells compared to the WT controls. *P < 0.01, unpaired *t*-test, *n* = 9 (5 mice) for WT group and *n* = 11 (5 mice) for L100P group. Scale bars, 100 pA and 200 ms. **d** I/O relationship between the fiber volley amplitude and fEPSP slope in hippocampal SC-CA1 synapses. Each point represents the mean of all slices tested for certain stimulus intensity. Error bars illustrate standard error for both axes. WT and L100P slices exhibit similar average I/O slope. *P* > 0.05, unpaired *t*-test, *n* = 24 (6 mice) for WT group and *n* = 18 (5 mice) for L100P group. **e** Paired-pulse ratio across different interpulse intervals in hippocampal SC-CA1 synapses. L100P group shows reduced paired-pulse facilitation. Two-way repeated measure ANOVA and Bonferroni post-tests, **P < 0.01 at interval of 100 ms, *n* = 12 slices from 5 mice for each group. **f** LTP induced by 100Hz (1 s) stimulus in hippocampal SC-CA1 synapses. fEPSP slopes normalized to the average baseline response (10 min) before LTP induction (time 0), are plotted in 1-min blocks. Sample traces show responses during baseline and the last 10 min of the recording (average of 10 recording traces). Scale bars, 0.1 mV and 4 ms. **g** Averaged percentage change of fEPSP slope after LTP induction showing reduced PTP (first 5 min of recording) and LTP (last 10 min of recording) in L100P mutant hippocampal SC-CA1 synapses. *P < 0.05, unpaired *t*-test, *n* = 21 slices from 6 WT mice, *n* = 14 slices from 5 L100P mutant mice. All data are shown as means ± SEM.

Since DISC1 is present in several cellular compartments including synapses, where it may interact with synaptic proteins to mediate synaptic function [24, 25], we decided to test whether *DISC1* L100P mutant mice showed dysregulation of synaptic transmission and plasticity in hippocampus, which may underlie its recognition deficits. First, we loaded hippocampal slices from juvenile mice (P8 ~ P15) with the fluorescent Ca^2+^ indicator Fluo-4 AM and monitored the changes of intracellular [Ca^2+^] after glutamate perfusion. We found that, although 50 μM glutamate challenge induced significant elevation of cytoplasmic [Ca^2+^] in both CA1 pyramidal cells and dentate gyrus granule cells of L100P hippocampal slices (Fig. 3; one-sample *t*-test, *P* < 0.05 or *P* < 0.01 compared to zero), the [Ca^2+^] elevation were much smaller than that observed in WT slice (Fig. 3c and d; unpaired *t*-test, *P* < 0.05 for CA1 cells and *P* < 0.01 for DG cells). Those results indicated that *DISC1* L100P mutation impaired neuronal activity in hippocampus. In order to determine the synaptic nature of impaired [Ca^2+^] increase in L100P mutants, we performed both evoked fEPSPs recording and basal postsynaptic currents (PSCs) recording on DG granule cells and CA1 pyramidal neurons from adult hippocampal slices. Dentate granule cells from L100P mice showed significant decrease in both sEPSCs frequency and sEPSCs amplitude compared to the WT controls (Fig. 4a; unpaired *t*-test; *t* = 3.09, *P* < 0.01 for sEPSCs frequency; *t* = 4.06, *P* < 0.001 for sEPSCs amplitude). Consistently, mEPSCs frequency and mEPSCs amplitude were also reduced in granule cells of L100P mice (Fig. 4b; unpaired *t*-test, *P* < 0.05 compared to the WT controls). Neither sIPSCs nor mIPSCs were changed in L100P mutant granule cells. However, only reduced sIPSCs amplitude was observed on CA1 pyramidal neurons carrying L100P mutation (Fig. 4c; unpaired *t*-test, *t* = 2.44, *P* < 0.05 in comparison to WT neurons); the sIPSCs frequency (Fig. 4c), sEPSCs and mEPSCs frequency and amplitude (data not shown) were not altered in CA1 pyramidal neurons of L100P hippocampus. Therefore, our data indicated the existence of abnormal, imbalanced glutamatergic and GABAergic transmission in hippocampal networks of L100P mutant mice.

Notably, fEPSPs recording in the SC-CA1 synapses of L100P mice hippocampal slices revealed normal basal synaptic transmission since its synaptic input/output (I/O) relationship was comparable to that of WT (Fig. 4d, average slope, 5.32 ± 0.359 for L100P mutants and 5.01 ± 0.325 for WT; unpaired *t*-test, *P* > 0.05). However, paired-pulse ratio was suppressed in CA1 of L100P mice hippocampus especially when interpulse interval was 100 ms, meaning impaired paired-pulse facilitation (Fig. 4e; two-way repeated measure ANOVA, genotype *F*_*(1, 110)*_ = 5.71, *P* < 0.05; Bonferroni post-tests, *P* < 0.01 at interval of 100 ms). Since paired-pulse facilitation (PPF) is a form of short-term plasticity caused by increased presynaptic Ca^2+^ concentration leading to a greater release of synaptic vesicles, the suppression of PPF indicates the existence of presynaptic abnormality in L100P mice hippocampus. Supportively, we also found suppression of post-tetanic potentiation (PTP), another form of short-term synaptic enhancement mediated by presynaptic activation of protein kinases, in SC-CA1 synapses of L100P mutant hippocampus (Fig. 4f and g, unpaired *t*-test; *t* = 2.49, *P* < 0.05 compared to WT, measured at 0–5 min after tetanic stimulation to induce LTP). Finally, LTP induced by high-frequency (100 Hz) stimulation in SC-CA1 synapses was also impaired in L100P hippocampal slices (Fig. 4f and g, unpaired *t*-test; t = 2.21, *P* < 0.05, measured at 50–60 min after tetanic stimulus). Because of PPF and PTP abnormality, both pre- and post-synaptic mechanisms may underlie the LTP deficits observed in L100P hippocampal slices. In contrast, MPP-DG synapses in L100P hippocampus displayed normal basal transmission, normal PPF and LTP induced by four trains of high-frequency stimulation (data not shown).

These results collectively suggest that *DISC1* L100P mutation leads to distinct changes of synaptic transmission and plasticity in two different hippocampal regions, both of which may contribute to the cognitive deficits observed in those mutant mice.

### L100P hippocampal neurons exhibited normal expression of excitatory synaptic markers and normal dendrites morphology

**Fig. 5:**
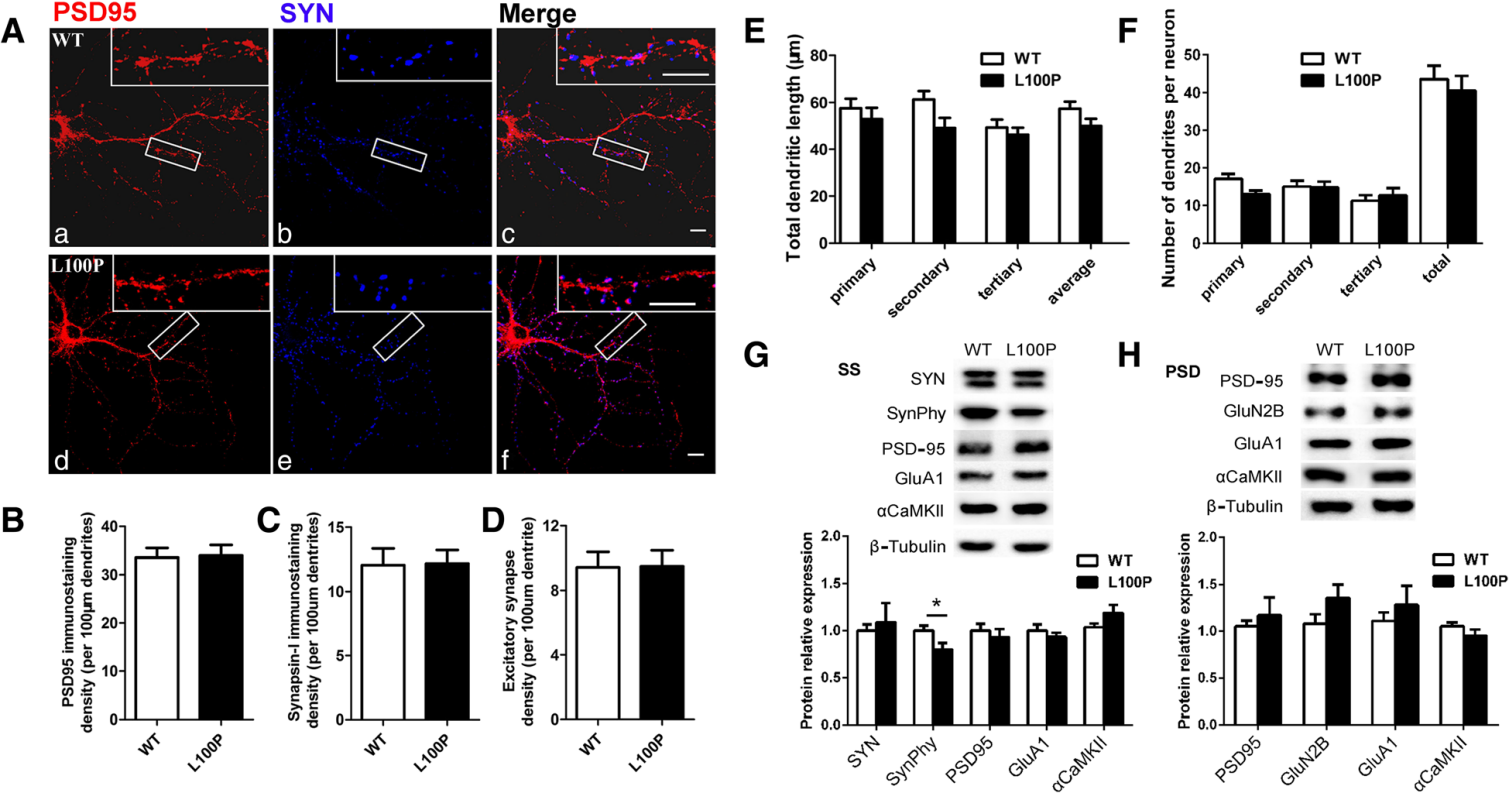
L100P hippocampal neurons exhibit normal expression of excitatory synaptic markers and dendrites morphology. **a** Immunostaining for PSD-95 (*red*) and synapsin-1(SYN, *blue*) in primary neuronal cultures from WT (a-c) and L100P (d-f) hippocampus. Scale bar figures, 50 μm; scale bar blow-out, 10 μm. **b**-**d** Summary of immunostaining density for PSD-95 (**b**), synapsin-1 (**c**) and excitatory synapse formation (**d**) in primary cultured L100P and WT hippocampal neurons. Spine density was calculated on secondary and tertiary dendrites. Unpaired *t*-test, *n* = 21 neurons for WT group and *n* = 24 neurons for L100P group. All data are shown as means ± SEM. **e**-**f** Summary histogram showing that L100P and WT neurons have similar total dendrites length (**e**) and dendrites number (**f**) in primary, secondary and tertiary dendritic segments. Two-way ANOVA and Bonferroni post-tests. WT group, *n* = 18 neurons; L100P group, *n* = 21 neurons. Data were collected from three to four independent experiments. All data are shown as means ± SEM. **g**-**h** Immunoblots showing synaptic proteins expression in the forebrain of WT mice and L100P mutants. Immunoblots of synaptosome (SS) fraction (**g**) and PSD-fraction (**h**) with antibodies against synapsin-1, synaptophysin, PSD-95, GluA1, αCaMKII, GluN2B and β-tubulin. *Upper*, representative sample immunoblot. β-tubulin is used to control for protein loading. *Lower*, summary of synaptic proteins expression in SS and PSD fractions. For quantification, the amount of each protein is first standardized as a ratio of protein/tubulin and then normalized by average protein/tubulin ratio of WT group before statistical analysis. Quantifications are based on the average of 6–12 independent experiments. *P < 0.05, unpaired *t*-test. All data are shown as means ± SEM.

To establish the molecular mechanisms that underlie the synaptic dysfunction in L100P mutants, we first measured the expression of PSD-95 and synapsin-1, two excitatory synaptic marker proteins, in primary cultured hippocampal neurons by immunohistochemistry techniques. Our analysis revealed that the basal expression of PSD-95 and synapsin-1 were not changed in L100P neurons (Fig. 5a-c). Consistently, our western blot analysis using mice forebrain synaptosome (SS) and PSD-enriched fractions indicated that the protein levels of PSD-95 and synapsin-1 in L100P mice were comparable to those measured in WT controls (Fig. 5g and h). Besides PSD-95 and synapsin-1, the expression level of other synaptic proteins including GluA1, GluN2B and αCaMKII were also comparable in two groups of mice (Fig. 5g and h). However, the expression of synaptophysin, the major synaptic vesicle protein p38, was reduced in SS-enriched fraction of L100P mice forebrain tissue (Fig. 5g, unpaired *t*-test; *t* = 2.92, *P* < 0.05), which may associate with pre-synaptic dysfunction observed in L100P mice hippocampus.

To test whether mutant hippocampal neurons exhibited dendrite structure or morphology abnormalities associated with synaptic dysfunction in L100P mice, we counted the number of PSD-95-immunopositive dendritic spines that co-localized with synapsin-1-postive boutons in primary cultured hippocampal neurons, and found no difference between L100P and WT neurons in the density of excitatory synapse (Fig. 5d). In addition, MAP2 staining showed that the total dendrite length and dendritic branches of cultured L100P neurons were similar to that of WT neurons (Fig. 5e and f, two-way ANOVA and Bonferroni post-tests, *P* > 0.05 for all segment levels comparison), indicating that L100P mutation did not dramatically change dendrite structure or morphology.

## Discussion

We reported here that ENU-induced *DISC1* L100P mutant shows certain recognition memory deficits, including both novel object recognition and object-place recognition, which is reversed by clozapine, but not haloperidol. Clozapine is an atypical antipsychotic that has antagonistic effect on multiple receptors, including 5-HT_2_, DA_1,_ M1, DA_4_ and DA_2_ [26]; while haloperidol is a typical antipsychotic which strongly blocks DA_2_ receptor. Previous studies indicated that clozapine and haloperidol regulates dopamine receptors function through different pathway and mechanisms [27]. Consistent with previous reports [4, 13], we did not find behavioral abnormality of L100P mutants in anxiety, depression, spatial learning and memory, and social ability. Also of note, we did not find hyperactivity phenotype of L100P mutants in novel open field or elevated plus maze test. Therefore, our data suggested that recognition memory deficits observed in L100P mutants were not caused by alterations in locomotor activity, visual-perceptual ability, anxiety or depression.

DISC1 is present in a broad of brain regions during development and adulthood, many of those brain regions are known to be abnormal in schizophrenia, such as the prefrontal cortex, hippocampus, and thalamus. Accumulating evidence indicates that DISC1 acts as an intracellular scaffold protein that in concert with numerous interacting proteins regulates neurogenesis, neuronal migration, neurite outgrowth in development brain and synaptic function in adulthood [24, 28–31]. Recent findings suggest that dysregulation of synaptic function and plasticity in neocortex and hippocampus are related to cellular and behavioral alterations observed in neuropsychiatric disorders such as schizophrenia and depression [1, 32, 33]. It is well accepted that recognition memory depends on a network of brain regions working in concert which include the perirhinal cortex, the medial prefrontal cortex, the hippocampus and the medial dorsal thalamus [34]. In particular, recognition memory concerning the spatial location of a previously encountered item involves the hippocampus [35]. Very recent study reported that object-in-place associative recognition memory depends on glutamate receptor neurotransmission within hippocampal-cortical circuits [36]. Therefore, we hypothesized that the object recognition deficits observed in *DISC1* L100P mice is associated with dysregulation of synaptic transmission and/or plasticity in hippocampus. Indeed, we found impairments in glutamate-evoked cytoplasmic [Ca^2+^] elevation in L100P mutant hippocampal slices suggesting reduced neuronal activity. Whole-cell patch-clamp recordings on granule cells further revealed a decrease in excitatory synaptic transmission while unchanged inhibitory synaptic transmission in L100P dentate gyrus, where granule cells receive major excitatory input from layer II of the entorhinal cortex and form the first connection of the hippocampal tri-synaptic loop circuit. In addition, fEPSPs recording disclosed both short-term and long-term plasticity deficits in the SC-CA1 hippocampal synapses of L100P mutant mice. Interestingly, L100P mutant hippocampal slices displayed the substantial reductions of EPSCs frequency and amplitude in DG granule cells, in contrast with rather normal EPSCs in CA1 pyramidal neurons. Meanwhile, the suppression of both short-term and long-term potentiation in SC-CA1 synapse contrasts with relatively normal plasticity in MPP-DG synapses of L100P mutant hippocampus. Although further details remain to be studied, our present results collectively suggest that *DISC1* L100P mutation leads to distinct changes of synaptic transmission and plasticity in two different hippocampal regions, both of which may interfere declarative information processing and lead to the cognitive deficits observed in those mutant mice. To be noted, dentate gyrus is also notable as being one of brain structures known to have high rates of neurogenesis in adult [37]. In support of the important role of DISC1 in neurogenesis, we also found reduced neurogenesis (BrdU^+^ cells) in the hippocampus of L100P mutants (132 ± 9, *n* = 9) compared to that of WT controls (179 ± 12, *n* = 8, unpaired *t*-test, *P* < 0.01, data not shown), which is consistent with previous report [38]. Previous studies showed that dentate gyrus-specific knockdown or deletion of adult neurogenesis impairs object recognition memory [39, 40], therefore we suspect that reduced neurogenesis may also contribute to recognition memory deficits in L100P mice; however, the extent of contribution of reduced neurogenesis towards recognition memory deficits in L100P mutants is not certain yet considering the involvement of adult neurogenesis in cognition, stress and emotion [41]. Noticeably, we did not find any behavioral phenotype of L100P mutants in locomotor activity, anxiety, depression, spatial learning and memory.

It was reported that the ENU-generated L100P mice have a single nucleotide mutation that affects the binding region of DISC1 with PDE4b and GSK3β [4, 12]. However, in our study, we did not find dramatic changes in GSK3β phosphorylation in L100P hippocampus lysates, nor did we find PDE4b inhibitor rolipram’s reversal of recognition deficits in L100P mice (data not shown). Although DISC1 regulation of neurite outgrowth in developing brain, and morphological differences such as reduced brain volume has been reported in L100P mutants [4], we did not find alterations in dendrites morphology (length and branch) or excitatory synapse density in primary cultured hippocampal cells derived from L100P mutant mice. Except for reduced synaptophysin expression detected in synaptosome (SS)-enriched fraction, we did not find significant changes of PSD-95, synapsin-1 and other synaptic proteins (CaMKII, GluA1, GluN2B and etc.) expressed in both cultured hippocampal cells and forebrain tissues derived from L100P mutants. Lack of changes in dendrites structure or DISC1 binding partner (i.e.GSK3β) activity may correlate to normal spontaneous activity observed in our L100P mice. It also suggests that the object recognition deficits exhibited by L100P mice in our study may result from glutamate and receptor dysfunction, for example AMPA and NMDA receptors activity in hippocampal network. Further studies are needed to elucidate the underlying molecular mechanisms.

## Conclusions

In conclusion, we report here that DISC1 L100P homozygous mutant shows impaired object recognition. The existence of abnormal synaptic transmission and plasticity in hippocampal network may disrupt declarative information processing and contribute to recognition deficits in DISC1 L100P mutant mice.

## Abbreviations

ACSF: Artificial cerebral spinal fluid
DAPI: 4′, 6-diaminodino-2-phenylindole
DG: Dentate gyrus
*DISC1*: Disrupted-in-schizophrenia 1
E18: 18-day embryo
ENU: *N*-ethyl-*N*-nitrosourea
EPM: Elevated plus maze
fEPSPs: Field excitatory postsynaptic potentials
FST: Forced swimming test
LTP: Long-term potentiation
mEPSCs: Miniature excitatory postsynaptic currents
mIPSCs: Miniature inhibitory post-synaptic currents
MPP: Medial perforant path
NOR: Novel object recognition
NPR: Object-place recognition
OF: Open field
PBS: Phosphate-buffered saline
PFA: 4 % paraformaldehyde
PPF: Paired-pulse facilitation
PSD: Post-synaptic density
PTP: Post-tetanic potentiation
ROIs: The regions of interest
SC: Schaffer collateral path
sEPSCs: Spontaneous excitatory postsynaptic currents
SI: Social interaction test
sIPSCs: Spontaneous inhibitory post-synaptic currents
SS: synaptosome
